# A new species of *Lepidepecreum* Spence Bate & Westwood, 1868 (Crustacea, Amphipoda, Tryphosidae) from the Clarion-Clipperton Zone in the abyssal east Pacific

**DOI:** 10.3897/zookeys.1274.127368

**Published:** 2026-03-24

**Authors:** Bronisław Wróblewski, Anna M. Jażdżewska

**Affiliations:** 1 Department of Invertebrate Zoology and Hydrobiology, Faculty of Biology and Environmental Protection, University of Lodz, Banacha 12/16, 90-237 Lodz, Poland University of Lodz Lodz Poland

**Keywords:** Amphipods, deep-sea mining, depth record, DNA barcoding

## Abstract

The Tryphosidae is a species-rich family of the order Amphipoda. During the survey of the abyssal depths of the Clarion-Clipperton Zone in the east Pacific, a new species of the genus *Lepidepecreum* was found. The species is here illustrated and described in detail; a confocal laser scanning microscope image and a molecular barcode are also provided. *Lepidepecreum myla***sp. nov**. differs from its congeners by the presence of a dorsal carina on all pereonites, moderately cleft telson (1/3 of the length), and a weak distal expansion of P7 basis, not extending longer than mid-merus. This study provides the first record of the genus from abyssal depths.

## Introduction

The amphipod family Tryphosidae, erected by Lowry and Myers (2017) with 43 described genera and 390 species, is a highly species rich family (Horton et al. 2024). The most species rich genera of this family are: *Tryphosella* Bonnier, 1893 (67 described species), *Hippomedon* Boeck, 1871 (58 spp.), *Orchomenella* Sars, 1890 (40 spp.) and *Lepidepecreum* Spence Bate & Westwood, 1868 (38 spp.) (Horton et al. 2024). Species of the genus *Lepidepecreum* are characterised by the possession of a mid-lateral bulge resulting in diamond-shaped appearance of the body cross-section, elongate peduncular article 3 of antenna 2 and a long carpus of gnathopod 1 (Lowry and Stoddart 2002b). The genus *Lepidepecreum* is mostly known from shallow-water records from all oceans; only five species, *Lepidepecreum baudini* Lowry & Stoddart, 2002, *Lepidepecreum californiensis* G. Vinogradov, 1994, *Lepidepecreum clypodentatum* J.L. Barnard, 1962, *Lepidepecreum gurjanovae* Hurley, 1963, *Lepidepecreum tourville* Lowry & Stoddart, 2002, have been recorded from depths below 1000 m (Barnard 1962; Hurley 1963; Vinogradov 1994; Lowry and Stoddart 2002b).

Study of the Amphipoda collected from abyssal depths in the Clarion-Clipperton Zone (CCZ), the area prospected for deep-sea mining, revealed the presence of a new species of *Lepidepecreum* which is illustrated and described here in detail. The cytochrome *c* oxidase (COI) barcode is also provided for further help in species identification.

## Material and methods

### Specific methodology

Material for the present study was sampled in the central-east Pacific, specifically in the easternmost sector of Clarion-Clipperton Zone (CCZ). The material was collected with an epibenthic sledge (EBS) during the ABYSSLINE-2 cruise (ABYSSal baseLINE project; Smith et al. 2015). For details of gear deployment and sample processing, see Jażdżewska and Horton (2026) and Jażdżewska et al. (2025).

The specimen was initially examined using a Leica M125 and a Nikon SMZ800 stereomicroscopes. The habitus of the holotype is presented as a photograph obtained with a confocal laser scanning microscope (CLSM). The holotype was stained in Congo red and acid fuchsin, temporarily mounted onto slides with glycerine and examined with a Leica TCS SPV equipped with a Leica DM5000 B upright microscope and three visible-light lasers (DPSS 10 mW 561 nm; HeNe 10 mW 633 nm; Ar 100 mW 458, 476, 488 and 514 nm), combined with the software LAS AF 2.2.1 (Leica Application Suite, Advanced Fluorescence). A series of photographic stacks was obtained, collecting overlapping optical sections throughout the whole preparation (Michels and Büntzow 2010; Kamanli et al. 2017).

Afterwards the specimen was dissected and mounted on permanent slides using polyvinyl-lactophenol containing lignin pink. All slides were examined using a Nikon Eclipse Ci compound microscope equipped with a camera lucida. Pencil drawings from the microscope were used as the basis for line drawings. The drawings were inked with Adobe® Illustrator® following the recommendations of Coleman (2003, 2009).

The following abbreviations were used in the figures: **A1, 2** = antenna 1, 2; **G1, 2** = gnathopod 1, 2; **LL** = lower lip; **Md** = mandible; **Mx1, 2** = maxilla 1, 2; **Mxp** = maxilliped; **P3–7** = pereopod 3–7; ; **U1–3** = uropod 1–3; **UL** = upper lip; **T** = telson; **l** = left; **r** = right.

The registered type material is deposited in the Senckenberg Museum, Frankfurt, Germany (**SMF**).

The individual was subjected to cytochrome *c* oxidase subunit I gene (COI) barcoding prior to identification of the species. The molecular procedures are described in Jażdżewska et al. (2025). The sequence was deposited in Barcode of Life Data Systems (BOLD, Ratnasingham and Hebert 2007) and in GenBank with the accession number: PQ734340. The relevant voucher information, taxonomic classification and sequence are deposited in the data set “DS-AMPHICCZ” in BOLD (https://doi.org/10.5883/DS-AMPHICCZ) (www.boldsystems.org).

## Results

### Systematics

**Order AMPHIPODA Latreille, 1816**

**Suborder AMPHILOCHIDEA Boeck, 1871**

**Superfamily LYSIANASSOIDEA Dana, 1849**

**Family TRYPHOSIDAE Lowry & Stoddart, 1997**

#### Genus Lepidepecreum

Taxon classificationAnimaliaAmphipodaTryphosidae

Spence Bate & Westwood, 1868

221D9489-6CD3-5D64-A6D4-4D5C696D95B1

Lepidepecreum Spence Bate & Westwood, 1868: 509

##### Type species.

*Lepidepecreum carinatum* Spence Bate & Westwood, 1868 accepted as *Lepidepecreum longicorne* (Spence Bate & Westwood, 1861) (type by subsequent designation).

##### Included species.

*Lepidepecreum* contains 38 species:

*Lepidepecreum alectum* Gurjanova, 1962,

*Lepidepecreum andamanensis* Lowry & Stoddart, 2002,

*Lepidepecreum baudini* Lowry & Stoddart, 2002,

*Lepidepecreum californiensis* G. Vinogradov, 1994,

*Lepidepecreum cingulatum* K.H. Barnard, 1932,

*Lepidepecreum clypeatum* Chevreux, 1888,

*Lepidepecreum clypodentatum* J.L. Barnard, 1962,

*Lepidepecreum comatum* Gurjanova, 1962,

*Lepidepecreum crenulatum* Chevreux, 1925,

*Lepidepecreum crypticum* Ruffo & Schiecke, 1977,

*Lepidepecreum dampieri* Lowry & Stoddart, 2002,

*Lepidepecreum eoum* Gurjanova, 1938,

*Lepidepecreum flindersi* Lowry & Stoddart, 2002,

*Lepidepecreum foraminiferum* Stebbing, 1888,

*Lepidepecreum freycineti* Lowry & Stoddart, 2002,

*Lepidepecreum garthi* Hurley, 1963,

*Lepidepecreum gurjanovae* Hurley, 1963,

*Lepidepecreum hirayamai* Lowry & Stoddart, 2002,

*Lepidepecreum infissum* Andres, 1983,

*Lepidepecreum kasatka* Gurjanova, 1962,

*Lepidepecreum longicorne* (Spence Bate & Westwood, 1861),

*Lepidepecreum lukini* (Budnikova, 1999),

*Lepidepecreum madagascarensis* Ledoyer, 1986,

*Lepidepecreum magdalenensis* (Shoemaker, 1942),

*Lepidepecreum myla* sp. nov. Wróblewski & Jażdżewska, 2025,

*Lepidepecreum nautilus* Gurjanova, 1962,

*Lepidepecreum rostratum* Gurjanova, 1962,

*Lepidepecreum sagamiensis* Gamo, 1975,

*Lepidepecreum serraculum* Dalkey, 1998,

*Lepidepecreum serratum* Stephensen, 1925,

*Lepidepecreum somchaii* Lowry & Stoddart, 2002,

*Lepidepecreum subclypeatum* Ruffo & Schiecke, 1977,

*Lepidepecreum takeuchii* Lowry & Stoddart, 2002,

*Lepidepecreum tourville* Lowry & Stoddart, 2002,

*Lepidepecreum twalae* Griffiths, 1974,

*Lepidepecreum typhlops* Bonnier, 1896,

*Lepidepecreum umbo* (Goës, 1866),

*Lepidepecreum urometacarinatum* Andres, 1985,

*Lepidepecreum vitjazi* Gurjanova, 1962.

##### Diagnosis

(after Barnard and Karaman 1991). Mouthparts forming quadrate bundle. Labrum and epistome differentially produced, prominent, separate, epistome strongly dominant in size and projection, blunt. Incisor ordinary, molar simple, small to medium, conicolaminate or subconical or setulose or smooth; palp attached strongly proximal to molar. Inner plate of maxilla 1 weakly (2) setose; palp bi-articulate, large. Inner and outer plates of maxilliped well developed, palp strongly exceeding outer plate, dactyl well developed. Coxa 1 slightly shortened and partly covered by coxa 2, (type) or not. Gnathopod 1 subchelate, palm oblique, articles 5 and 6 variable, either dominant, dactyl large; article 6 of gnathopod 2 slightly shorter than article 5, ordinary, propodus minutely subchelate. Inner ramus of uropod 2 without notch. Uropod 3 almost aequiramous, ordinary, peduncle scarcely elongate, outer ramus bi-articulate. Telson elongate, deeply cleft or rarely entire.

The single individual collected fully agrees with the generic diagnosis so there is no doubt of its taxonomic assignation at this taxonomic level.

#### Lepidepecreum myla sp. nov.

Taxon classificationAnimaliaAmphipodaTryphosidae

C8B92A43-9A54-552D-87AB-724D3390F4AF

https://zoobank.org/A80604F4-0B5F-415E-BD02-3272A2D34346

Figs 1, 2, 3, 4, 5

##### Type material.

***Holotype***: Pacific • SMF 63357, immature female, 8.5 mm, Clarion-Clipperton Zone; UKSR-1 exploration contract area, R/V Thompson, ABYSSLINE-2, AB2-EB03, 23/02/2015, 12°33.78'N, 116°37.5'W, 4219 m, COI: PQ734340.

**Figure 1.**
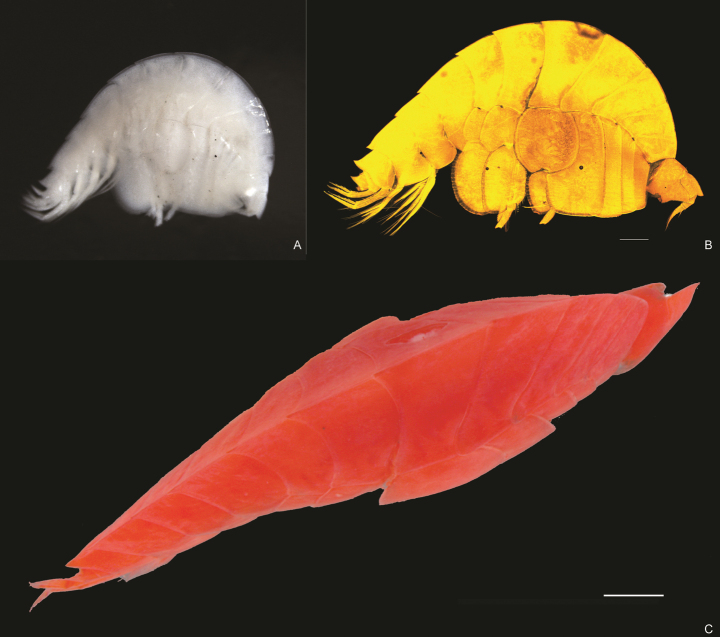
*Lepidepecreum myla* sp. nov. **A**. Photograph of unstained individual before manipulation; **B**. CLSM photography; **C**. Dorsal view of the animal (stained for CLSM photography), holotype female, 8.5 mm, SMF 63357. Scale bars: 0.5 mm.

**Figure 2.**
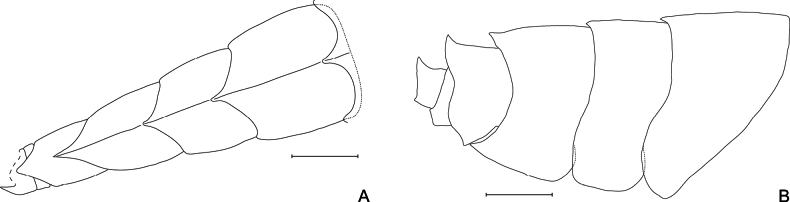
*Lepidepecreum myla* sp. nov. **A**. Dorsal view of pleosome and urosome; **B**. Side view of pleosome and urosome, holotype female, 8.5 mm, SMF 63357. Scale bars: 0.5 mm.

**Figure 3.**
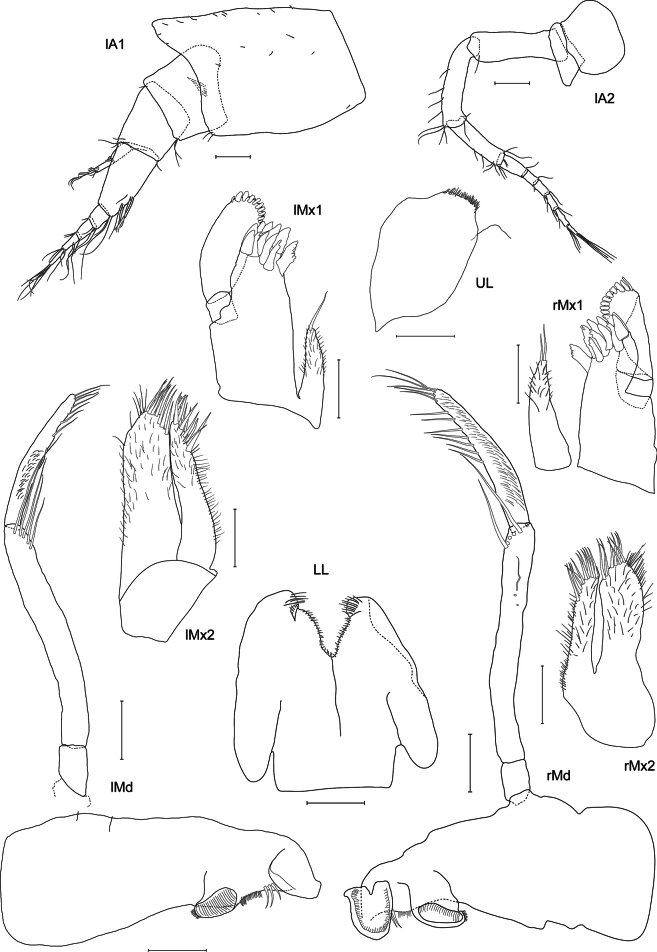
*Lepidepecreum myla* sp. nov. holotype female, 8.5 mm, SMF 63357. Scale bars: 0.1 mm (**A1, A2, LL, Md, Mx1; Mx2, UL**).

**Figure 4.**
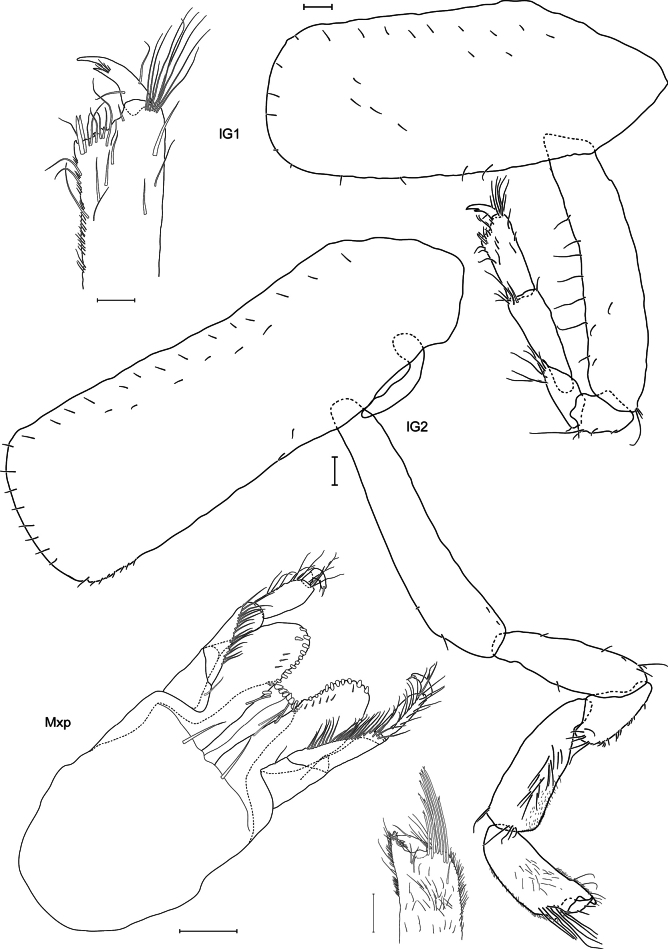
*Lepidepecreum myla* sp. nov. holotype female, 8.5 mm, SMF 63357. Scale bars: 0.1 mm (**G1, G2, Mxp**).

**Figure 5.**
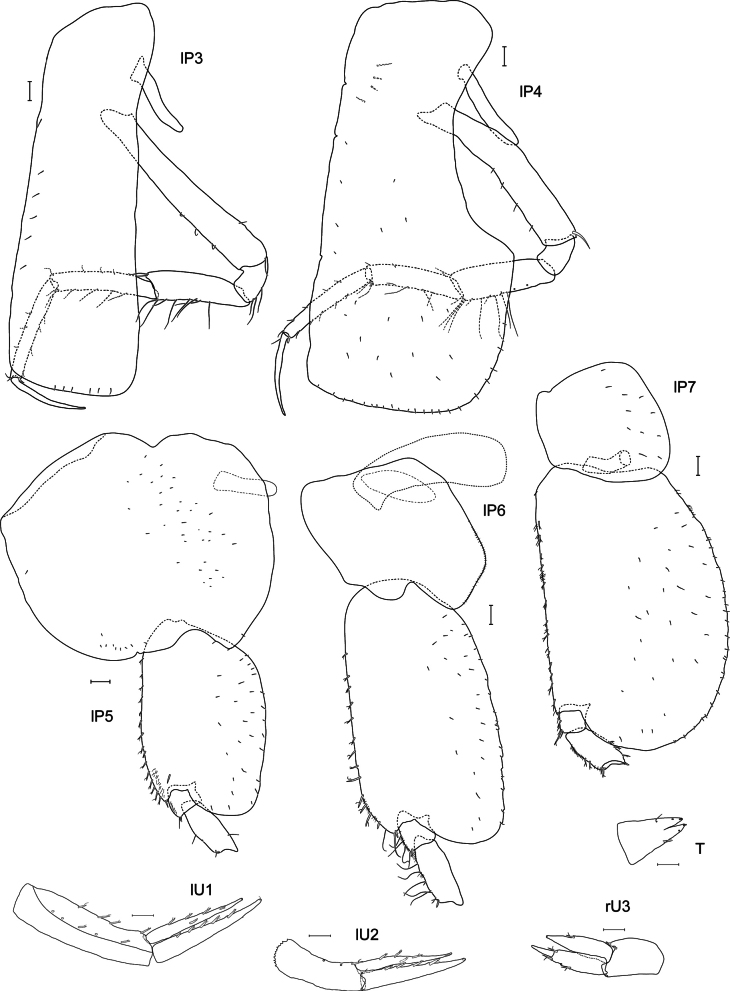
*Lepidepecreum myla* sp. nov. holotype female, 8.5 mm, SMF 63357. Scale bars: 0.1 mm (**P3, P4, P5, P6, P7, U1, U2, U3, T**).

##### Type locality.

Abyssal Pacific Ocean, Clarion-Clipperton Zone, 12°33.78'N, 116°37.5'W, 4219 m.

##### Etymology.

This species is named for Myla, the character from the computer video game Hollow Knight. The representatives of the described species, like Myla, are just little arthropods trying to survive in total darkness. The name is used as noun in apposition.

##### Diagnosis.

Body with dorsal carina present along all pereonites, pleonites and urosomite 1; antenna 1 article 1 with anterodistal lobe; pereopod 5–7 merus not or only slightly expanded; basis P7 with posterodistal lobe extending up to 0.65 length of merus; telson cleft 1/3.

##### Description.

Based on holotype immature female, 8.5 mm, registration number SMF 63366.

***Body*** (Figs 1, 2): robust, with mid-lateral bulge resulting in diamond-shaped appearance in cross-section; dorsal carina present along all pereonites, pleonites and urosomite 1; pereonites 6–7 and pleonites 1–3 dorsally produced to a single tooth (teeth progressively longer with the last one reaching 1/4 of the urosomite 1). ***Coxae***: coxae 1–4 deeper than long, coxa 1 smaller but not hidden by coxa 2, coxae 2 and 3 of similar size and shape (coxa 2 slightly smaller), coxa 4 the largest, coxa 5 1/2 depth of coxa 4, coxae 6 and 7 progressively smaller. ***Urosomite 1*** (Figs 1, 2): dorsally with a large, upturned tooth. ***Urosomite 2*** (Figs 1, 2): dorsally smooth. ***Urosomite 3*** (Figs 1, 2): dorsally smooth but with pair of teeth dorso-laterally. ***Epimeron 1*** (Figs 1, 2): evenly rounded. ***Epimeron 2*** (Figs 1, 2): anteriorly rounded, posteriorly subquadrate. ***Epimeron 3*** (Figs 1, 2): anteriorly rounded, posteriorly extending into sharp tooth.

***Head*** (Fig. 1): deeper than long, as long as pereonite 1; rostrum vestigial.

***Lateral cephalic lobe*** (Fig. 1): large, reaching the end of article 1 of antenna 1, subtriangular with tip rounded.

***Eye*** (Fig. 1A): the trace of the eye visible as white ovoid spot on the head.

***Antenna 1*** (Figs 1, 3): short, as long as head and pereonites 1 and 2 combined, length ratio of peduncular articles 1–3 1:0.4:0.4; article 1 with large anterodistal subtriangular lobe (tip rounded); article 2 wider than article 3, sparse setae at article edges and some setules on the surface; primary flagellum five-articulate, with sparse setae placed distally on flagellar articles, first article weakly callynophorate, slightly longer than peduncular article 3, accessory flagellum short, tri-articulate, with sparse setae placed distally.

***Antenna 2*** (Fig. 3): short, subequal in length to antenna 1; peduncular article 1 moderate size, rounded, article 2 small with small blunt gland cone reaching 1/8 length of peduncle article 3, article 3 subequal in length to article 4, article 5 shorter, relative lengths of peduncle articles 3–5 1:1:0.7, sparse setae placed distally on articles 3–5, four setae on anterior edge of article 4; flagellum six-articulate, with sparse setae distally on flagellar articles.

***Upper lip*** (Fig. 3): anteriorly rounded with anterior margin covered with short setules.

***Mandible*** (Fig. 3): incisor margin smooth blade-like; accessory spine row with three (left) or two (right) spines; molar columnar with oval, fully triturating surface; palp attached proximally, tri-articulate, longer than mandible body, articles 1–3 in ratio of 1:5.1:3.4, article 2 with five setae distally, article 3 slightly curved and narrowing distally, inner margin with six (left) or seven (right) setae and three setae apically.

***Lower lip*** (Fig. 3): inner and outer lobes rounded apically, covered with fine setae, mandibular lobes large, rounded.

***Maxilla 1*** (Fig. 3): inner plate small, reaching to the basis of palp article 1 and narrow, slightly tapering distally, rounded with one (left) or two (right) apical setae, covered with fine setules; outer plate with eleven dentate setal-teeth; palp bi-articulate, article 1 0.3× length of article 2, slender; article 2 wider than article 1, with nine (left) or ten (right) spines apically.

***Maxilla 2*** (Fig. 3): inner plate more slender and shorter than outer, with eleven setae apically; outer plate with nine setae apically.

***Maxilliped*** (Fig. 4): inner plate subrectangular reaching 1/2 length of palp article 2 with three strong spines and two setae apically; outer plate long, reaching 1/3 palp article 3, inner margin with one long (basal) and ten short spines, additional spines on the surface; palp (twisted on both sides, exact proportions not checked) with progressively narrowing articles, articles 1–3 of similar length, article 4 the shortest, inner margin of articles 2–3 with row of long setae, article 4 with single short seta apically.

***Gnathopod 1*** (Fig. 4): subchelate; coxa smaller than coxa 2, subrectangular, length ~ 2× width, anterior margin straight, anteroventral corner rounded, short setules on the surface; basis long, length 4.8× width, seven thin setae along anterior margin; ischium subrectangular, posteroventral corner with a seta; merus trapezoidal, posterodistal margin with a group of setae; carpus long, length 3.5× width, with group of setae posterodistally; propodus subrectangular, length 0.7× carpus, fine setae along posterior margin and moderately long and slender setae in the distal 1/2 of the joint, palm slightly oblique, with two spines at palmar corner and a row of seven setae along palm; dactylus short, length 0.4× propodus, incised at 1/3 from the end, one seta on anterior and posterior margin and a group of setules at the incision.

***Gnathopod 2*** (Fig. 4): minutely chelate; coxa subrectangular, length 3.3× width, anterior and ventral margins straight, short setules on the surface and along distal margin, posterodistal corner with single setule; basis long, length 5.7× width, slightly widening distally; ischium long, 0.5× length of basis, sparse setae on both margins; merus triangular, with setules along posterodistal margin; carpus long, length 2.9× width, setulose on the surface and posterior margin; propodus 0.8× length of carpus, length 2.4× width, setulose on the surface and both margins and a group of long setae anterodistally, palm projecting distally, its tip with four short, stouter setae; dactylus short, length 0.3× propodus, with one seta in the middle of anterior margin and a few setae at posterior margin.

***Pereopod 3*** (Fig. 5): coxa subrectangular, length 3.3× width, slightly widening distally, short setules on the surface and along distal margin; basis long and narrow, slightly widening distally, length 7× width, anterior margin with three setae, posterior margin with one seta, posterodistal corner with one seta; ischium subrectangular, posterior margin with three setae; merus not widening distally, two setae at anterodistal margin, six setae along posterior margin, posterodistal corner with two setae; carpus slender, length 6× width, anterior margin with three short setae, anterodistal corner with one short seta, posterior margin with four setae, posterodistal corner with two setae; propodus narrower than carpus, length 8× width, anterior margin with three short setae, anterodistal corner with two short setae, posterior margin with four short setae, posterodistal corner with one stouter seta; dactylus long and slender; articles 2–7 length proportions: 1:0.2:0.4:0.5:0.5:0.4.

***Pereopod 4*** (Fig. 5): coxa subrectangular with large posterodistal lobe, short setules on the surface and along distal margin; remaining articles as for pereopod 3 except for a few more setae at merus, carpus and propodus; articles 2–7 length proportions: 1:0.2:0.5:0.5:0.6:0.4.

***Pereopod 5*** (Fig. 5): coxa very large, bilobed, rounded, slightly wider than deep (width 1.1× depth); basis expanded, length 1.3× width, anterior margin with 16 stout, short setae, posterior margin rounded, smooth with posterodistal rounded lobe reaching 0.4 length of merus; merus not expanded, slightly produced along carpus, sparse setae on anterior and posterior margins; carpus–dactylus broken off, articles 2–4 length proportions: 1:0.1:0.4.

***Pereopod 6*** (Fig. 5): coxa smaller than coxa 5, slightly wider than deep (width 1.1× depth), bilobed, posterolobate, anterior lobe small; basis expanded, length 1.7× width, anterior margin with 16 stout, short setae, posterior margin rounded, with small crenulations armed with fine setae, posterodistal rounded lobe reaching 0.25 length of merus; merus not expanded, slightly produced along carpus, six long setae along anterior margin; carpus–dactylus broken off, articles 2–4 length proportions: 1:0.1:0.3.

***Pereopod 7*** (Fig. 5): coxa smaller than coxa 6, subrectangular, almost as wide as deep, basis expanded, length 1.4× width, anterior margin with 14 stout, short setae, posterior margin rounded, almost entirely smooth, armed with fine setae, posterodistal rounded lobe reaching 0.65 length of merus; merus slightly expanded, slightly produced along carpus, sparse short and stout setae along anterior and posterior margins; carpus–dactylus broken off, articles 2–4 length proportions: 1:0.1:0.2.

***Uropod 1*** (Fig. 5): lanceolate; peduncle longer than rami, dorsolateral and dorsomedial margins with five and four spines respectively; inner ramus 0.7× length of peduncle, dorsolateral and dorsomedial margins with five and four spines, respectively (some spines broken off); outer ramus length 1.2× inner ramus, dorsomedial margin with five spines; inner ramus dorsolateral and dorsomedial margin with two and three small spines, respectively.

***Uropod 2*** (Fig. 5): lanceolate; shorter than uropod 1; peduncle subequal in length to inner ramus, three spines on dorsomedial margin; inner ramus dorsolateral and dorsomedial margins with one and three spines respectively; outer ramus length 1.2× inner ramus, dorsomedial margin with four spines.

***Uropod 3*** (Fig. 5): lanceolate; peduncle length 0.8× inner ramus, only three short spines at distal end of peduncle; inner ramus 0.9× length of outer ramus, dorsomedial margin with one spine, outer ramus bi-articulate, 2^nd^ article 0.4× length of the 1^st^, dorsolateral and dorsomedial margins of the 1^st^ article with one and three spines, respectively.

***Telson*** (Fig. 5): subtriangular, length 2× width, cleft 30%, lobes narrowing, distodorsal margin with two setae and two short spines on each lobe.

##### Intraspecific variation.

No other specimens to compare.

##### Molecular identification.

Following the definition given by Pleijel et al. (2008), the sequence of the holotype female of *L. myla* (SMF 63357, GenBank accession number PQ734340) is designated as a hologenophore. The species has also received a Barcode Index Number from Barcode of Life Data Systems: BOLD:AEB0340 (https://doi.org/10.5883/BOLD:AEB0340).

##### Remarks.

The genus *Lepidepecreum* consists of a group of species characterised by the combination of a diamond-shaped body cross-section resulting from the presence of mid-lateral bulge, an elongate peduncular article 3 on antenna 2 and a long carpus on gnathopod 1. The presently described species fully matches the above criteria. The genus encompasses 38 described species. Table 1 summarises main differences of *L. myla* sp. nov. and the remaining species of the genus. The majority of species within the genus (33 species) have a telson cleft 50% or deeper. Only a single species in the genus, *L. infissum*, is known to have an entire telson. *Lepidepecreum myla* sp. nov. may be distinguished easily from these species by the cleft of the telson which is 30% of the length (Table 1). Species of the genus with a similar telson cleft of 25–30% include: *L. flindersi*, *L. freycineti*, *L. tourville*, and *L. urometacarinatum*. Among them *L. flindersi*, *L. freycineti*, and *L. tourville* are characterised by expanded merus of pereopod 5–7 that is not expanded in *L. myla* sp. nov. Additionally, *L. flindersi* and *L. freycineti* possess a very large posterodistal lobe on basis of pereopod 7 that extends beyond merus (not extending deeper than 1/3 of the merus length in *L. myla* sp. nov.). From *L. tourville* the new species differ also by the dorsal carina that is present on all pereonites, pleonites and urosomite 1 (vs present on pleonites 1–3 in *L. tourville*), the shape of postero-distal corner of epimeral plate 3 being formed as a small tooth in the new species (vs subquadrate in *L. tourville*) and article 2 of uropod 3 being 0.4 length of the 1^st^ (vs article 2 being only 0.3 length of the 1^st^ in *L. tourville*). *Lepidepecreum myla* sp. nov. shares many characters with *L. urometacarinatum*, however it differs from this species by (characters of *L. urometacarinatum* provided in brackets): telson cleft 30% (vs 25%), head interantennal lobe distally rounded (vs distally acute), dorsal carina present on all pereonites, pleonites and urosomite 1 (vs present on pleonite 3 only), dorsal teeth present on pereonites 6 and 7, pleonites 1–3, and urosomite 1 (vs absent).

**Table 1.** Table summarising main characters differentiating *Lepidepecreum myla* sp. nov. from known species with information about their geographic and bathymetric distribution. Character states that are shared between known species and newly described taxon are given in bold.

**Species/Character**	**Telson cleft**	**Antenna 1, peduncle article 1**	**Antenna 1, peduncle article 2**	**Head lobe**	**Eyes (present /absent)**	**Merus of P5-7**	**Basis of P7 posterodistal lobe**	**Dorsal carina**	**Dorsal teeth**	**Epimeral plate 3**	**Depth [m]**	**Region**	**Location**	**Reference**
*Lepidepecreum alectum* Gurjanova, 1962	3/4	**produced into rounded lobe**	**not produced**	**distally rounded**	present	expanded	**not extending beyond merus**	on pleonite 3 and urosomite 1	on pleonite 3 and urosomite 1	subacute tooth	129	NW Pacific	E of Zelyony Island, Kuril Islands	Gurjanova 1962
*Lepidepecreum andamanensis* Lowry & Stoddart, 2002	3/4	not produced	**not produced**	distally subacute	absent	expanded	extending up to mid carpus	on pleonite 3 only	on pleonite 3 and urosomite 1	extended backwards but not in the form of acute tooth	61	Indian Ocean	Phuket Island (Thailand)	Lowry and Stoddart 2002a
*Lepidepecreum baudini* Lowry & Stoddart, 2002	1/2	not produced	**not produced**	distally subacute	absent	expanded	extending up to the end of carpus	on pleonites 2 and 3	on pleonites 2, 3 and urosomite 1	**acutely produced**	1840	SW Pacific	Point Hicks, Australia	Lowry and Stoddart 2002b
*Lepidepecreum californiensis* G. Vinogradov, 1994	3/4	**strongly produced**	**not produced**	distally acute	absent	**slightly expanded**	**not extending beyond merus**	on pereonites 5-7, pleonites and urosomite 1	on pereonites 5-7, pleonites and urosomite 1	rounded	2779	NE Pacific	Monterey Bay	Vinogradov 1994
*Lepidepecreum cingulatum* K.H. Barnard, 1932	1/2	not produced	unknown	distally subrectangular	present	expanded	**not extending beyond merus**	absent	absent	narrowly rounded	0-320	Antarctic	South Orkney Islands, Antarctic Peninsula	Barnard 1932; De Broyer et al. 2007
*Lepidepecreum clypeatum* Chevreux, 1888	> 3/4	**produced**	**not produced**	distally acute	absent	expanded	extending up to the end of carpus	present	on pleonite 3 and urosomite 1	**with tooth**	180	NE Atlantic	Isle of Groix, Bay of Biscay	Chevreux 1888
*Lepidepecreum clypodentatum* J.L. Barnard, 1962	> 3/4	**produced into rounded lobe**	**not produced**	distally subacute	absent	unknown	extending up to the end of carpus	on pleonites 1-3	on pleonites 1-3 and urosomite 1	**with tooth**	1861	SE Atlantic	Cape Basin	Bonnier 1896
*Lepidepecreum comatum* Gurjanova, 1962	2/3	**acutely produced**	**not produced**	**distally rounded**	present	expanded	**not extending beyond merus**	**on all pereonites, pleonites and urosomite 1**	on pereonites 3-7, pleonites 1-3 and urosomite 1	narrowly rounded	40-500	NW Pacific	Shikotan Island, Kuril Basin, Sea of Japan, Sakhalin, Promorie, Sea of Okhotsk	Gurjanova 1962
*Lepidepecreum crenulatum* Chevreux, 1925	3/4	not produced	**not produced**	distally subacute	present	expanded	extending almost up to the end of carpus	absent	on pleonite 3 and urosomite 1	narrowly rounded	80	NE Atlantic	Coast of Sahara	Chevreux 1925
*Lepidepecreum crypticum* Ruffo & Schiecke, 1977	3/4	**produced into rounded lobe**	**not produced**	large, distally rounded	present	expanded	**not extending beyond merus**	not pronounced	absent, pleonite 3 and urosomite 1 with rounded boss	rounded	4-10	Mediterranean	Central Mediterranean	Ruffo and Schiecke 1977
*Lepidepecreum dampieri* Lowry & Stoddart, 2002	1/2	not produced	**not produced**	distally subacute	absent	expanded	extending up to mid propodus	on pleonite 3 only	on pleonite 3 and urosomite 1	subquadrate	120-122	Indian Ocean	Western Australia	Lowry and Stoddart 2002b
*Lepidepecreum eoum* Gurjanova, 1938	2/3	**produced into rounded lobe**	**not produced**	distally subacute	present	expanded	**not extending beyond merus**	on pereonites 5-7, pleonites and urosomite 1	pronounced boss on urosomite 1	rounded	15	NW Pacific	Sea of Japan	Gurjanova 1938
*Lepidepecreum flindersi* Lowry & Stoddart, 2002	**1/3**	not produced	produced into rounded lobe	**distally rounded**	absent	expanded	extending beyond carpus	on pleonite 3 only	on pleonite 3 and urosomite 1	subquadrate	120	SW Pacific	Flinders island (Australia)	Lowry and Stoddart 2002b
*Lepidepecreum foraminiferum* Stebbing, 1888	1/2	**produced**	**not produced**	long and narrow	absent	expanded	**not extending beyond merus**	**on all pereonites, pleonites and urosomite 1**	small on pleonites 1-3, large on urosomite 1	**with tooth**	232-716	Antarctic	Antarctic, Enderby Land, MacRobertson Shelf, Prydz Bay, sub-Antarctic: Kerguelen Island	Stebbing 1888; Lowry and Stoddart 2002b
*Lepidepecreum freycineti* Lowry & Stoddart, 2002	**1/3**	**produced into rounded lobe**	**not produced**	distally subacute	absent	expanded	extending beyond merus	on pleonite 3 only	on pleonite 3 and urosomite 1	narrowly rounded	600-800	SW Pacific	Tasman Sea, Freycinet Peninsula	Lowry and Stoddart 2002b
*Lepidepecreum garthi* Hurley, 1963	2/3	**acutely produced**	**not produced**	unknown	absent	**not expanded**	**not extending beyond merus**	**on all pereonites, pleonites and urosomite 1**	on pereonites 2-7, pleonites 1-3 and urosomite 1	subquadrate	320-402	NE Pacific	California	Hurley 1963
*Lepidepecreum gurjanovae* Hurley, 1963	3/4	**produced into rounded lobe**	**not produced**	unknown	present	expanded	**not extending beyond merus**	**on pereonites, pleonites and urosomite 1**	small on all segments of the body, large on urosomite 1	**with tooth**	256-1829	NE Pacific	West coast of USA (33-50° )	Hurley 1963
*Lepidepecreum hirayamai* Lowry & Stoddart, 2002	3/4	not produced	**not produced**	distally subacute	present	expanded	**not extending beyond merus**	absent	on urosomite 1 only	subquadrate	3-25	NW Pacific	East China Sea (west Kyushu Island)	Lowry and Stoddart 2002a
*Lepidepecreum infissum* Andres, 1983	entire	not produced	**not produced**	distally subacute	present	**slightly expanded**	**not extending beyond merus**	absent	absent	**with tooth**	113-765	Antarctic	Antarctica, Antarctic Peninsula, Amery Depression, MacRobertson Shelf, Prydz Bay	Andres 1983; Lowry and Stoddart 2002b
*Lepidepecreum kasatka* Gurjanova, 1962	3/4	not produced	**not produced**	distally subacute	present	expanded	**not extending beyond merus**	on pleonites 2, 3 and urosomite 1	on pleonites 2, 3 and urosomite 1	subquadrate	123-229	NW Pacific	Shikotan Island, Iturup Island, Kuril Islands	Gurjanova 1962
*Lepidepecreum longicorne* (Spence Bate, 1862)	> 3/4	**produced**	produced	**distally rounded**	present	expanded	**not extending beyond merus**	**on all pereonites, pleonites and urosomite 1**	on pleonite 3 and urosomite 1	subquadrate	0.3-70	N Atlantic, Mediterranean	British Isles, S Norway, Denmark, Mediterranean	Sars 1891
*Lepidepecreum lukini* (Budnikova, 1999)	2/3	**produced into rounded lobe**	**not produced**	**distally rounded**	present	expanded	**not extending beyond merus**	on urosomite 1 only	rounded boss on urosomite 1	subacute tooth	10-40	NW Pacific	Sakhalin, Moneron Island, Sea of Japan	Budnikova 1999
*Lepidepecreum madagascarensis* Ledoyer, 1986	1/2	not produced	**not produced**	**distally rounded**	present	expanded	extending almost up to the end of merus	on pleonite 3 and urosomite 1	on pleonite 3 and urosomite 1	rounded	15 and sublittoral	Indian	Nosy Be, Madagascar	Ledoyer 1986
*Lepidepecreum magdalenensis* (Shoemaker, 1942)	2/3	not produced	**not produced**	**distally rounded**	present	expanded	**not extending beyond merus**	absent	on urosomite 1 only	subquadrate	18-27	NE Pacific	MagdaIena Bay, California	Shoemaker 1942
***Lepidepecreum myla* sp. nov. Wróblewski & Jażdżewska, 2025**	**1/3**	**produced**	**not produced**	**distally rounded**	trace of eye visible as white spot	**not expanded**	**not extending beyond merus**	**on all pereonites, pleonites and urosomite 1**	**on pereonites 6-7, pleonites 1-3, urosomite 1**	**with tooth**	**4219**	**central E Pacific**	**Clarion-Clipperton Zone**	**present study**
*Lepidepecreum nautilus* Gurjanova, 1962	3/4	**produced into rounded lobe**	**not produced**	distally subquadrate with small point	present	expanded	**not extending beyond merus**	on pereonites 5-7, pleonites and urosomite 1	rounded boss on pleonite 1 and urosomite 1	subquadrate	150-414	NW Pacific	Shikotan Island, Sea of Okhotsk	Gurjanova 1962
*Lepidepecreum rostratum* Gurjanova, 1962	2/3	**acutely produced**	**not produced**	**distally rounded**	present	expanded	**not extending beyond merus**	on posterior pereonites and pleonite 1	on pereonite 7, pleonite 1 and urosomite 1	**with tooth**	414	NW Pacific	Shikotan Island	Gurjanova 1962
*Lepidepecreum sagamiensis* Gamo, 1975	3/4	not produced	**not produced**	**distally rounded**	present	expanded	**not extending beyond merus**	**on all pereonites, pleonites and urosomite 1**	on all pereonites, pleosomites and urosomite 1	rounded	704	NW Pacific	Sagami Bay, NW Pacific	Gamô 1975
*Lepidepecreum serraculum* Dalkey, 1998	3/4	**produced into rounded lobe**	**not produced**	**distally rounded**	present	expanded	**not extending beyond merus**	on pleonite 3 and urosomite 1	pronounced boss on urosomite 1	rounded	0-150	NE Pacific	California to Alaska	Dalkey 1998
*Lepidepecreum serratum* Stephensen, 1925	2/3	**acutely produced**	**not produced**	distally acute	absent	expanded	**not extending beyond merus**	on pereonites 5-7, pleonites and urosomite 1	on pereonites 5-7, pleonites and urosomite 1	rounded	320-900	NE Atlantic	SW Iceland, SW Faroes	Stephensen 1925
*Lepidepecreum somchaii* Lowry & Stoddart, 2002	3/4	**produced into rounded lobe**	produced into rounded lobe	distally subacute	present	expanded	**not extending beyond merus**	on urosomite 1 only	on urosomite 1 only	narrowly rounded	32-61	Indian	Phuket Island (Thailand)	Lowry and Stoddart 2002a
*Lepidepecreum subclypeatum* Ruffo & Schiecke, 1977	> 3/4	**acutely produced**	acutely produced	distally subacute	absent	expanded	extending beyond merus	absent	on pleonite 3 and urosomite 1	subquadrate	203	Mediterranean	Adriatic Sea	Ruffo and Schiecke 1977
*Lepidepecreum takeuchii* Lowry & Stoddart, 2002	3/4	**produced**	**not produced**	distally acute or subacute	present	expanded	**not extending beyond merus**	absent	pronounced rounded boss on urosomite 1	subquadrate	shallow waters (exact value not provided)	NW Pacific	Matsukawa-ura, Fukishima Prefecture, Japan	Lowry and Stoddart 2002a
*Lepidepecreum tourville* Lowry & Stoddart, 2002	**1/3**	**produced**	**not produced**	distally subacute	absent	expanded	**not extending beyond merus**	on pleonites 1-3	on pleonites 1-3 and urosomite 1	subquadrate	996-1850	SW Pacific	Tasman Sea, Cape Tourville	Lowry and Stoddart 2002b
*Lepidepecreum twalae* Griffiths, 1974	3/4	not produced	**not produced**	**distally rounded**	present	expanded	**not extending beyond merus**	absent	on urosomite 1 only	narrowly rounded	121	Indian	South of S Africa	Griffiths 1974
*Lepidepecreum typhlops* Bonnier, 1896	3/4	**produced into rounded lobe**	**not produced**	**distally rounded**	absent	unknown	**not extending beyond merus**	absent	on urosomite 1 only	unknown	650-950	NE Atlantic	bay of Biscay, SW Faroes	Bonnier 1896; Stephensen 1925
*Lepidepecreum umbo* (Goës, 1866)	> 3/4	**produced**	**not produced**	distally acute	present	**not expanded**	**not extending beyond merus**	present, more pronounced on posterior segments	**on pereonites 6-7, pleonites 1-3, urosomite 1**	rounded	20-630	NE Atlantic	N Norway, Iceland, Spitsbergen	Goës 1866; [Bibr B39][Bibr B45]
*Lepidepecreum urometacarinatum* Andres, 1985	< 1/4	**produced**	**not produced**	distally acute	present	**slightly expanded**	**not extending beyond merus**	on pleonite 3 only	absent	**with tooth**	100-806	Antarctic	Antarctica, Scotia Sea, Antarctic peninsula, Weddell Sea, off Enderby Land	Andres 1983, 1985; Havermans et al. 2018; Lowry and Stoddart 2002b
*Lepidepecreum vitjazi* Gurjanova, 1962	3/4	**produced into rounded lobe**	**not produced**	**distally rounded**	present	expanded	**not extending beyond merus**	on pleonite 3 and urosomite 1	on urosomite 1 only	**with tooth**	40	NW Pacific	Bering Sea	Gurjanova 1962

Five species of *Lepidepecreum* have been recorded at depths below 1000 m: *L. baudini* (1840–2500 m), *L. californiensis* (2779 m), *L. clypodentatum* (1861 m), *L. gurjanovae* (256–1829 m), and *L. tourville* (996–1850 m) (Barnard 1962; Hurley 1963; Vinogradov 1994; Lowry and Stoddart 2002b; Table 1). The current finding of *L. myla* sp. nov. at the depth of 4219 m provides the first abyssal and the deepest record of the genus worldwide.

Only two of all 38 described *Lepidepecreum* species have been barcoded: *L. gurjanovae* (sequence ID: BHAK663-18, not associated with a publication, only available in BOLD) and *L. urometacarinatum* (MH825748.1; Havermans et al. 2018). The molecular distance of *L. myla* sp. nov. to these two species exceeds 17% of p-distance (17.2% to *L. gurjanovae* and 17.4% to *L. urometacarinatum*). It is worth noting that also between the two formerly described species the p-distance reaches 15.7%. One could expect higher molecular similarity of *L. gurjanovae* and *L. myla* as both species were collected in the North East Pacific and the former species is recognised to inhabit bathyal depths (256–1829 m; Hurley 1963). However, it must be underlined that the barcoded individual identified as *L. gurjanovae* was collected in British Columbia at the depth of only 13 m and may be a misidentification.

## Supplementary Material

XML Treatment for Lepidepecreum

XML Treatment for Lepidepecreum myla

## Additional information

### Author ORCIDs

Bronisław Wróblewski https://orcid.org/0009-0005-1063-7085

Anna M. Jażdżewska https://orcid.org/0000-0003-2529-0641

### Data availability

All of the data that support the findings of this study are available in the main text.
